# Supplementary material for: Eyes on incivility in surgical teams: Teamwork, well-being, and an intervention
Source: PLoS One. 2023 Nov 30;18(11):e0295271. doi: 10.1371/journal.pone.0295271 (PMC10688855; doi:10.1371/journal.pone.0295271)
Supplement: Attachment — Submitted filename: Response to Reviewers.docx [file pone.0295271.s002.docx]

Comments to Reviewers

Reviewer #1: Thanks for the opportunity to review this manuscript. The authors have done a great job at addressing reviewer comments. I just have several minor comments for the authors that would benefit the manuscript.

Introduction

The following sentence in paragraph 2 “To add to the growing body of literature on incivility in healthcare, we investigate relationships between individuals’ experiences and observations of incivility, team dynamics, and ultimately well-being using mediated analytical models.” would be better located in the final paragraph of the introduction.

Thank you for your suggestion. As suggested, we have moved this sentence to the final paragraph.

The introduction could be strengthened with mention of the culture change programs in the US and Australia (Promoting Professional Accountability Program - Vanderbilt University Medical Centre, and in Australia - Ethos program). Although mentioned in the Discussion, it could be made clearer that these programs do aim to reduce low level / low intensity behaviours such as incivility in order to reduce the likelihood of patterns of unprofessional behaviour and the gradation to more serious behaviours such as physical or sexual assault. A key to these programs is the informal feedback process in which trained staff provide feedback to those reported for unprofessional behaviour to increase awareness and provide an opportunity for change. A majority of behaviours reported in these studies are incivility behaviours, and there is evidence to suggest these programs decrease incivility. It is worth mentioning this in the introduction.

We appreciate your comment and have added some further information on these programs to the introduction, highlighting that these interventions have shown a positive impact on the reduction of disruptive behaviors in the healthcare space. A limitation of these programs are the additional costs and resources needed for these systems to be successfully implemented, justifying the need for potential low-costs options (such as the eye signage) to be explored.

Materials and Methods

Please include the survey items as an appendix. I think this would be beneficial for the reader and those who may want to replicate the study.

We did not initially include the survey items as an appendix as our items are all derived from previously used measures. However, in response we have now made the items publicly available at <https://doi.org/10.17605/OSF.IO/67HNV>

and have indicated this in the text.

“Signs were of 2 sizes (210 x 297cm and 297 x 420cm).” Do you mean mm rather than cms? 420cm is 4 meters.

Thank you for noticing this oversight on our behalf. We have changed this to mm.

Results

Please provide a citation given that your approach to examining the open ended question was thematic analysis.

A citation has now been added: Braun V, Clarke V. Using thematic analysis in psychology. Qual Res Psychol. 2006 Jan 1;3(2):77-101.
